# Correlation Between Non-insulin-Based Insulin Resistance Indices and Increased Arterial Stiffness Measured by the Cardio–Ankle Vascular Index in Non-hypertensive Chinese Subjects: A Cross-Sectional Study

**DOI:** 10.3389/fcvm.2022.903307

**Published:** 2022-07-05

**Authors:** Xin Zhang, Runyu Ye, Chaoping Yu, Tianhu Liu, Xiaoping Chen

**Affiliations:** ^1^Department of Cardiology, West China Hospital, Sichuan University, Chengdu, China; ^2^Department of Cardiology, Pidu District People’s Hospital, The 3rd Affiliated Hospital of Chengdu Medical College, Chengdu, China

**Keywords:** arterial stiffness, insulin resistance, cardio-ankle vascular index, TG/HDL-C, TyG, MEST-IR

## Abstract

Data are limited on the relationship between the cardio–ankle vascular index (CAVI) and non-insulin-based insulin resistance (IR) indices, including the triglyceride to high-density lipoprotein cholesterol ratio (TG/HDL-C), fasting triglyceride and glucose index (TyG), and metabolic score for IR (METS-IR). In this study, we explored the relationship between TG/HDL-C, TyG, METS-IR, and the risk of increased arterial stiffness (CAVI ≥ 8.0) and compared their ability to detect arterial stiffness in the non-hypertensive Chinese population. A total of 3,265 non-hypertensive subjects were included. Spearman’s and partial correlation analyses were used to assess the relationship between non-insulin-based IR indices and CAVI. The correlation between these indices and the risk of a CAVI ≥ 8.0 was explored by multiple logistic regression analysis. The area under the receiver-operating characteristic curve was used to compare the ability of TG/HDL-C, TyG, and METS-IR to detect a CAVI ≥ 8.0. After adjustment for confounding factors, linear regression analysis showed that the CAVI changed by 0.092 [95% confidence interval (CI) 0.035–0.149] per standard deviation increase in TyG. While, this linear relationship was not found when analyzing TG/HDL-C and METS-IR. Multiple logistic regression analysis showed that the proportion of patients with CAVI ≥ 8.0 in the fourth quartile of TG/HDL-C [Q4 vs. Q1: odds ratio (OR) 2.434, 95% CI 1.489–3.975], TyG (Q4 vs. Q1: OR 2.346, 95% CI 1.413–3.896), and METS-IR (Q4 vs. Q1: OR 2.699, 95% CI 1.235–5.897) was significantly higher than that in the lowest quartile. The area under the curve that could discriminate CAVI ≥ 8.0 was 0.598 (95% CI 0.567–0.629) for TG/HDL-C, 0.636 (95% CI 0.606–0.667) for TyG, and 0.581 (95% CI 0.550–0.613) for METS-IR. In this study, we demonstrated a significant association between increased arterial stiffness and non-insulin-based IR indices. Among them, TyG showed better discriminatory ability than TG/HDL-C or METS-IR.

## Introduction

Arterial stiffness isan age-related disorder that is characterized by loss of elastin fibers and an increase in collagen fibers in the media of the arteries (1) and is an independent predictor of the risk of cardiovascular disease (CVD), hypertension-mediated target organ damage, and CVD-related mortality (2, 3). Measurement of arterial stiffness has become a routine method for assessment of vascular health in the clinical setting. The most commonly used non-invasive assessment methods include the brachial–ankle pulse wavy velocity PWV (baPWV), carotid–femoral PWV (cfPWV), cardio–ankle vascular index (CAVI), arterial pressure–volume index, and arterial velocity–pulse index (4).

Insulin resistance (IR) is a common metabolic disorder in which the response of the tissues and organs to insulin is impaired, leading to poor oxidation of glucose and a reduction in glycogen synthesis (5). A previous study demonstrated that IR leads to arterial stiffness by causing hyperinsulinemia, dysfunction of endothelial cells, oxidative stress, and inflammation (6, 7). The gold standard method used to assess IR is the hyperinsulinemic-euglycemic clamp. However, this method is invasive, complex, and time-consuming (8) and not widely used for health screening purposes or in epidemiological studies. Recently, several non-insulin-based IR indices, including the triglyceride to high-density lipoprotein cholesterol ratio (TG/HDL-C) (9), fasting triglyceride and glucose index (TyG) (10), and metabolic score for IR (METS-IR) (11), have emerged and shown a good correlation with the hyperinsulinemic-euglycemic clamp method. Therefore, we now have a simple and reliable alternative biomarker of IR.

Several studies have demonstrated a relationship between higher values for these non-insulin-based IR indices and an increased risk of arterial stiffness as measured by baPWV (12–16). However, the majority of these studies were performed in hypertensive patients, community-dwelling elderly individuals, postmenopausal women, or apparently healthy populations in other countries. Moreover, the CAVI, which reflects arterial stiffness from the origin of the aorta to the ankle (17), is not affected by changes in blood pressure (BP) and is superior to baPWV for assessment of arterial stiffness (18, 19). However, evidence for a relationship between non-insulin-based IR indices and CAVI is lacking. Finally, none of the above-mentioned studies compared the ability of TG/HDL, TyG, or METS-IR to detect arterial stiffness. In this cross-sectional study, we explored the relationship between the TG/HDL-C, TyG, METS-IR, and arterial stiffness and compared the ability of these indices to assess the risk of increased arterial stiffness in non-hypertensive community-dwelling Chinese adults in Chengdu, Southwest China.

## Materials and Methods

### Study Participants

The study had a retrospective cross-sectional design and included adults who had attended for a wellness check in the Department of Physical Examination, Pidu District People’s Hospital, Chengdu, between January 2011 and December 2013. The following exclusion criteria were applied: previous history of hypertension and average office BP ≥ 140/90 mmHg at the time of the health screen; on hypoglycemic or lipid-lowering medication; pregnancy; malignancy; myocardial infarction, angina, heart failure, or atrial fibrillation; disability involving the lower limbs; and an ankle-brachial index of <0.9. Nine patients in whom the CAVI was abnormally high (>13) or low (<4) were also excluded. Finally, data for 3,265 individuals were available for analysis. The study was approved by the regional ethics committee and conducted according to the principles of the Declaration of Helsinki.

### Physical Examination and Collection of Medical Information

Well-trained investigators collected information on basic demographics, lifestyle factors, past medical history, and any medication for chronic disease using a standard questionnaire. Physical examination (height, weight, BP, and CAVI) was performed in a quiet room with an ambient temperature of approximately 25°C. Office BP was measured using an electronic sphygmomanometer (HEM-7200, Omron, Kyoto, Japan) after 5 min of rest. Systolic and diastolic BP were obtained three times in the right arm in a sitting position. The average of the three readings was used for the final analyses.

### Measurement of Biological Parameters

Blood samples were collected from each subject in the morning after 10–12 h of overnight fasting. Plasma fasting glucose (FPG), triglycerides (TGs), total cholesterol (TC), high-density lipoprotein cholesterol (HDL-C), low-density lipoprotein cholesterol (LDL-C), uric acid (UA), and creatinine were measured by an automatic biochemical analyzer in the Department of Laboratory Medicine, Pidu District People’s Hospital, Chengdu.

### Measurement of Cardio–Ankle Vascular Index

The CAVI was measured automatically in all cases using non-invasive equipment (VaSera VS-1000VS-1000; Fukuda Denshi Co., Ltd., Tokyo, Japan) on the same day as the physical examination and blood tests. In all cases, measurements were obtained after 15 min of rest in the supine position. Cuffs were wrapped around both upper arms and ankles. Electrocardiography electrodes were attached to both wrists. A microphone was placed at the left fourth rib near the sternum to record a phonocardiogram. After approximately 5 min of testing, the values for CAVI and other parameters were obtained from an embedded printer. The CAVI was calculated as follows:


(1) CAVI=a{(2ρ/(Ps--Pd))×ln(Ps/Pd)PWV}2+b

where *a* and *b* are constants, ρ is blood density, *Ps* is systolic BP (SBP), *Pd* is diastolic BP (DBP), and PWV is pulse wave velocity (20). The average of the left and right CAVI values was used in the final analyses.

### Definitions

Patients were deemed to have hypertension if they had an average SBP ≥ 140 mmHg and/or an average DBP ≥ 90 mmHg, were on antihypertensive therapy, or had a preexisting diagnosis of hypertension. Prehypertension was defined as an SBP of 120–139 mmHg and/or a DBP of 80–89 mmHg without antihypertensive medication (21). Diabetes mellitus (DM) was defined according to previous diagnosis or an FPG ≥ 7.0 mmol/L (22). Hyperuricemia was defined as a serum UA level ≥ 420 μmol/L in men and ≥360 μmol/L in women (23). Renal dysfunction was defined as an increase in creatinine to ≥115 μmol/L according to the upper limit of the reference value defined by the local laboratory. Body mass index (BMI) was calculated as kg/m^2^. Smoking status was classified as “current non-smoker” or “current smoker” according to self-reported information. Similarly, alcohol consumption status was classified as “current drinker” (drinking more than once a month) and “current non-drinking status” (“former drinker,” or “never or almost never drinker”). A CAVI < 8.0 was considered normal based on the criteria for physiological diagnosis recommended by the vascular failure committee in Japan (24). In this study, we defined a CAVI ≥ 8.0 as a borderline or abnormal increase in arterial stiffness. The non-insulin-based IR indices were calculated as follows:

TG/HDL-C = TG (mg/dL)/HDL-C (mg/dL)(9); TyG = Ln [fasting TG (mg/dL) × FPG (mg/dL)/2] (10); and METS-IR = Ln [(2 × fasting FPG (mg/dL)) + TG (mg/dL)] × BMI/(Ln [HDL-C (mg/dL)]) (11).

### Statistical Methods

Continuous data with a normal distribution are expressed as the mean ± standard deviation (SD) and those with an abnormal distribution are shown as the median (interquartile range). Categorical variables are expressed as the frequency (percentage). Continuous data were compared using the independent-samples *t*-test, Mann–Whitney *U*-test, or Kruskal–Wallis test. Differences in categorical variables were compared among the groups using the chi-squared test. Bivariate Spearman’s and partial correlation analyses were used to detect the relationship between each non-insulin-based IR index and CAVI. We also standardized the non-insulin-based IR indices and entered them into linear regression models to explore the association between the increases in TG/HDL per SD, TyG per SD, METS-TR per SD, and CAVI. The positive relationship between TyG and CAVI was then validated using a generalized additive model with smoothness of fit. The three non-insulin-based IR indices (TG/HDL-C, TyG, and METS-IR) were also divided into quartiles, and the lowest quartile was used as a reference. This categorization was performed as follows: quartile 1 (Q1): ≤ 0.503, quartile 2 (Q2): 0.504–0.760, quartile 3 (Q3): 0.761–1.217, and quartile 4 (Q4): ≥ 1.218 for TG/HDL-C; Q1: ≤ 7.980, Q2: 7.981–8.331 Q3: 8.332–8.759, and Q4: ≥ 8.760 for TyG; and Q1: ≤ 27.241, Q2: 27.242–30.837, Q3: 30.838–35.504, and Q4: ≥ 35.505 for METS-IR. The relationship between the quartiles for each non-insulin-based IR index and increased arterial stiffness (CAVI ≥ 8.0) was then examined by multiple logistic regression analysis after adjusting for confounding factors. Finally, the ability to detect increased arterial stiffness (CAVI ≥ 8.0) was compared between TG/HDL-C, TyG, and METS-IR using receiver-operating characteristic (ROC) curve analysis. Furthermore, the optimal cutoff value, Youden index (YI), sensitivity, and specificity were calculated for each index. All statistical analyses were performed using SPSS version 26.0 (IBM Corp., Armonk, NY, United States) or EmpowerStats (X&Y Solutions, Inc., Boston, MA, United States). A *P*-value < 0.05 was considered statistically significant.

## Results

### Demographic and Clinical Data

**FIGURE 1:**
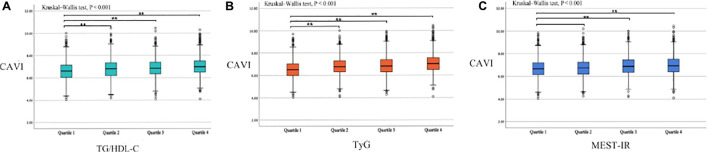
Box plots showing the CAVI value according to each non-insulin-based insulin resistance index quartile. **(A)** TG/HDL-C quartiles; **(B)** TyG quartiles; **(C)** METS-IR quartiles. Center lines show the medians, box limits indicate the 25th and 75th percentiles, whiskers extend the interquartile range from the 25th to 75th percentiles 1.5 times, and outliers are represented by dots. CAVI, cardio–ankle vascular index; TG/HDL-C, triglyceride to high-density lipoprotein cholesterol ratio; TyG, fasting triglyceride and glucose index; METS-IR, metabolic score for insulin resistance. **p < 0.05.

Table 1 shows the baseline subject demographics and clinical characteristics according to whether CAVI was < 8.0 or ≥ 8.0. Subjects with a CAVI ≥ 8.0 were older, had higher BMI, SBP, DBP, FPG, UA, creatinine, TG, TC, and LDL-C values, and were more likely to be male, smokers, and consumers of alcohol, and to have prehypertension and DM history. There was no statistically significant between-group difference in height, weight, HDL-C level, or the proportion with hyperuricemia or renal dysfunction. We found that the CAVI increased with increasing non-insulin-based IR index values. The median CAVI (interquartile range) values were 6.60 (6.05–7.15), 6.80 (6.20–7.35), 6.85 (6.35–7.40), and 7.00 (6.50–7.50) for TG/HDL-C quartiles (*P* < 0.001), 6.50 (6.00–7.05), 6.80 (6.30–7.30), 6.85 (6.30–7.40), and 7.05 (6.50–7.55) for TyG quartiles (P < 0.001), and 6.65 (6.15–7.20), 6.75 (6.20–7.25), 6.90 (6.35–7.45), and 6.95 (6.40–7.50) for METS-IR quartiles (P < 0.001). The relevant information is shown in Figure 1.

**TABLE 1:** Baseline characteristics of the individuals included in the analysis.

Variables	Whole cohort *N* = 3,265	CAVI < 8.0 *N* = 2,944	CAVI ≥ 8.0 *N* = 321	*P*-value
Age, years	40.15 ± 12.34	38.18 ± 10.72	58.24 ± 11.41	<0.001
Male (%)	47.0% (1534/3265)	44.6% (1313/2944)	68.8% (221/321)	<0.001
Smoking (%)	24.7% (808/3265)	23.4% (690/2944)	36.8% (118/321)	<0.001
Drinking (%)	34.4% (1123/3265)	33.7% (993/2944)	40.5% (130/321)	0.014
Pre-HTN (%)	36.5% (1192/3265)	34.4% (1014/2944)	55.5% (178/321)	<0.001
DMself-report (%)	1.2% (36/3265)	0.5% (15/2944)	6.5% (21/321)	<0.001
HUA (%)	17.3% (565/3265)	16.9% (499/2944)	20.6% (66/321)	0.104
RD (%)	1.2% (39/3265)	1.1% (33/2944)	1.9% (6/321)	0.241
Height (cm)	163.19 ± 7.75	163.23 ± 7.76	162.85 ± 7.68	0.415
Weight (Kg)	60.03 ± 10.50	59.96 ± 10.57	60.72 ± 9.88	0.217
BMI (Kg/m^2^)	22.45 ± 2.93	22.41 ± 2.93	22.83 ± 2.88	0.015
SBP (mmHg)	117.79 ± 9.53	117.31 ± 9.38	122.17 ± 9.83	<0.001
DBP (mmHg)	76.11 ± 6.76	75.81 ± 6.76	78.85 ± 6.11	<0.001
FPG (mg/dL)	83.17 (77.75–90.41)	83.17 (77.75–90.41)	86.79 (79.56–97.64)	<0.001
UA (mg/dL)	5.35 (4.43–6.44)	5.29 (4.39–6.38)	5.75 (4.98–6.86)	<0.001
CREA (mg/dL)	0.87 (0.76–1.01)	0.86 (0.75–1.00)	0.96 (0.85–1.06)	<0.001
TG (mg/dL)	99.20 (71.74–143.49)	97.43 (69.97–139.86)	116.92 (85.03–169.62)	<0.001
TC (mg/dL)	164.86 (145.95–186.35)	163.71 (145.17–184.56)	175.68 (154.05–199.04)	<0.001
HDL-C (mg/dL)	56.76 (48.26–65.73)	56.76 (48.47–66.02)	55.60 (46.72–65.06)	0.056
LDL-C (mg/dL)	86.49 (71.81–104.63)	85.04 (71.01–103.47)	94.59 (81.24–111.78)	<0.001
TG/HDL-C	0.76 (0.50–1.22)	0.74 (0.49–1.18)	0.90 (0.63–1.58)	<0.001
TyG	8.33 (7.98–8.76)	8.31 (7.96–8.73)	8.57 (8.24–8.99)	<0.001
METS-IR	30.84 (27.24–35.51)	30.59 (27.12–35.30)	32.63 (29.22–36.70)	<0.001
CAVI	6.85 ± 0.90	6.65 ± 0.69	8.62 ± 0.58	<0.001

*Pre-HTN, prehypertension; DM, diabetes mellitus; HUA, hyperuricemia; RD, renal dysfunction; BMI, body mass index, SBP, systolic blood pressure; DBP, diastolic blood pressure; FPG, fasting plasma glucose; UA, Uric Acid; CREA, creatinine; TG, triglyceride; TC, total cholesterol; HDL-C, high density lipoprotein cholesterol; LDL-C, low density lipoprotein cholesterol; TyG, triglyceride and glucose index; METS-IR, metabolic score for insulin resistance; CAVI, cardio–ankle vascular index.*

### Correlations Between Non-insulin-Based Insulin Resistance Indices and Cardio–Ankle Vascular Index

Spearman correlation analysis (Table 2) was used to detect the relationship between each non-insulin-based IR index and CAVI. TG/HDL-C (*r* = 0.189, *P* < 0.001), TyG, (*r* = 0.223, *P* < 0.001), and METS-IR (*r* = 0.139, *P* < 0.001) were all significantly related to CAVI. After adjusting for age, sex, BMI, SBP, and DBP, all the non-insulin-based IR indices were still correlated with CAVI, and the partial correlation coefficients were 0.078 for TG/HDL-C, 0.114 for TyG, and 0.109 for METS-IR.

**TABLE 2:** Spearman and partial correlation between non-insulin-based IR indices and CAVI.

Variables	CAVI			
	Spearman correlation		Partial correlation	
	r	*p*	*r*	*p*
TGı/HDL-C	0.189	<0.001	0.078	<0.001
TyG	0.223	<0.001	0.114	<0.001
METS-IR	0.139	<0.001	0.109	<0.001

*TG, triglyceride; HDL-C, high density lipoprotein cholesterol; TyG, triglyceride and glucose index; METS-IR, metabolic score for insulin resistance; CAVI, cardio-ankle vascular index.*

*Age, sex, BMI, SBP, and DBP were controlled in partial correlation analysis.*

### Association Between Non-insulin-Based Insulin Resistance Indices and Cardio–Ankle Vascular Index

**FIGURE 2:**
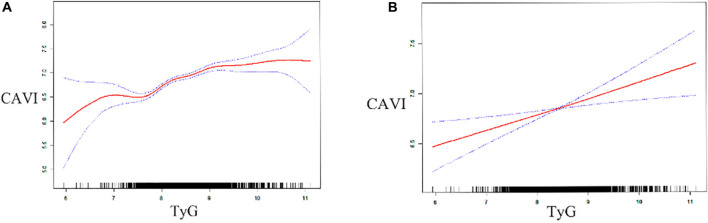
Generalized additive model plot showing the relationship between TyG and CAVI. Data unadjusted **(A)** and adjusted **(B)** for age, sex, body mass index, smoking status, alcohol consumption, systolic and diastolic blood pressure, pre-hypertension, diabetes mellitus, hyperuricemia, renal dysfunction, triglycerides, total cholesterol, high-density and low-density lipoprotein cholesterol, fasting plasma glucose, uric acid, and creatinine. CAVI, cardio–ankle vascular index; TyG, fasting triglyceride and glucose index.

After fully adjusting for confounding factors (model 4), including age, sex, BMI, smoking, alcohol consumption, prehypertension, DM, hyperuricemia, renal dysfunction, SBP, DBP, FPG, creatinine, UA, TG, TC, HDL-C, and LDL-C, linear regression analysis (Table 3) showed that CAVI had a change of 0.092 (95% CI 0.035–0.149) per SD increase in TyG. While, this linear relationship was not found when analyzing TG/HDL-C and METS-IR. Furthermore, a significant positive correlation between TyG and CAVI was found in a generalized additive model (Figure 2). Furtherly, the correlation per SD increase in each non-insulin-based IR index value and CAVI was then determined according to whether subjects were aged ≤ 40 or > 40 years (according to the average age of this cohort). A linear association was found between TyG (β 0.135, 95% CI 0.043–0.226) and CAVI only in subjects aged > 40 years (Table 4). Each non-insulin-based IR index was then divided into quartiles, with the lowest quartile used as the reference. After fully adjusting for confounding factors (model 4), the fourth quartiles for TG/HDL-C and TyG were significantly correlated with increases in CAVI of 0.155 (95% CI 0.043–0.266) and 0.171 (95% CI 0.071–0.271), respectively, when compared with the lowest quartile. However, the relationship between the quartiles of METS-IR and CAVI was not significant in the fully adjusted linear regression model (Table 3).

**TABLE 3:** Linear regression analyses for the association between non-insulin-based IR indices and CAVI.

Variables	β (95%CI), CAVI			
	Model 1	Model 2	Model 3	Model 4
**Per SD increase**				
TG/HDL-C	0.100 (0.069–0.130)**	0.055 (0.031–0.079)**	0.050 (0.026–0.074)**	0.001 (–0.053–0.056)
TyG	0.194 (0.164–0.224)**	0.081(0.056–0.107)**	0.068 (0.041–0.094)**	0.092 (0.035–0.149)*
METS-IR	0.106 (0.075–0.136)**	0.159(0.109–0.210)**	0.135 (0.082–0.187)**	0.121 (-0.031 to 0.272)
**TG/HDL-C**				
Q1	**Reference**	**Reference**	**Reference**	**Reference**
Q2	0.238 (0.152–0.323)**	0.100 (0.036–0.164)*	0.097 (0.033–0.161)*	0.086 (0.018–0.154)*
Q3	0.291(0.205–0.377)**	0.112 (0.046–0.179)*	0.107 (0.041–0.174)*	0.085 (0.005–0.165)*
Q4	0.442 (0.357–0.528)**	0.223 (0.151–0.295)**	0.206 (0.133–0.279)**	0.155 (0.043–0.266)*
**TyG**				
Q1	**Reference**	**Reference**	**Reference**	**Reference**
Q2	0.303 (0.218–0.388) **	0.134 (0.070–0.197)**	0.134 (0.070–0.198)**	0.131 (0.065–0.196)*
Q3	0.362 (0.277–0.447)**	0.112 (0.046–0.179)*	0.110 (0.043–0.176)*	0.098 (0.024–0.171)*
Q4	0.566 (0.481–0.650)**	0.236 (0.165–0.306)**	0.205 (0.132–0.278)**	0.171 (0.071–0.271)*
**METS-IR**				
Q1	**Reference**	**Reference**	**Reference**	**Reference**
Q2	0.080 (-0.006 to 0.167)	0.005 (-0.065 to 0.076)	-0.004 (-0.075 to 0.066)	-0.056 (-0.134 to 0.021)
Q3	0.241 (0.155–0.327)**	0.087 (0.001–0.174)*	0.067 (-0.021 to 0.154)	-0.036 (-0.142 to 0.070)
Q4	0.300 (0.213–0.386)**	0.215 (0.097–0.334)**	0.170 (0.049–0.219)*	-0.022 (-0.183 to 0.139)

*TG, triglyceride; HDL-C, high density lipoprotein cholesterol; TyG, triglyceride and glucose index; METS-IR, metabolic score for insulin resistance; CAVI, cardio-ankle vascular index; SD, standard deviation; CI, confidence interval.*

*Model 1: unadjusted.*

*Model 2: adjusted for age, sex, and BMI.*

*Model 3: adjusted for age, sex, BMI, smoking, drinking, prehypertension, diabetes mellitus, hyperuricemia, renal dysfunction, SBP, and DBP.*

*Model 4: adjusted for age, sex, BMI, smoking, drinking, prehypertension, diabetes mellitus, hyperuricemia, renal dysfunction, SBP, DBP, FPG, CREA, UA, TG, TC, HDL-C, and LDL-C.*

*p < 0.05; **P < 0.001.

**TABLE 4:** Linear regression analyses for the association between non-insulin-based IR indices and CAVI in age subgroup.

Variables		β (95%CI), CAVI	
		Model 1	Model 2
**Per SD increase**	**Age** ≤ **40 years old**		
TG/HDL-C		0.078 (0.048–0.108)**	-0.010 (-0.070 to 0.050)
TyG		0.114 (0.082–0.146)**	0.006 (–0.009 to 0.137)
METS-IR		0.039 (0.007–0.071)*	0.103 (-0.098 to 0.304)
**Per SD increase**	**Age** > **40 years old**		
TG/HDL-C		0.068 (0.018–0.117) *	-0.015 (-0.151 to 0.121)
TyG		0.132 (0.086–0.177) **	0.135 (0.043–0.226) *
METS-IR		0.048 (0.002–0.094) *	0.097 (-0.136 to 0.330)

*TG, triglyceride; HDL-C, high density lipoprotein cholesterol; TyG, triglyceride and glucose index; METS-IR, metabolic score for insulin resistance; CAVI, cardio-ankle vascular index; SD, standard deviation; CI, confidence interval.*

*Model 1: unadjusted.*

*Model 2: adjusted for age, sex, BMI, smoking, drinking, prehypertension, diabetes mellitus, hyperuricemia, renal dysfunction, SBP, and DBP; FPG, CREA, UA, TG, TC, HDL-C, and LDL-C.*

*p < 0.05; **P < 0.001.

### Association Between Non-insulin-Based Insulin Resistance Indices and a Cardio–Ankle Vascular Index ≥ 8.0

The association between each non-insulin-based IR index and a CAVI ≥ 8.0 was examined by logistic regression (Table 5). Setting the lowest quartile as a reference, univariate analysis showed that Q2, Q3, and Q4 of each non-insulin-based IR index (TG/HDL-C, TyG, and METS-IR) was significantly associated with a CAVI ≥ 8.0 in all subjects. After fully adjusting for confounding factors (including age, sex, BMI, smoking, drinking, prehypertension, diabetes mellitus, hyperuricemia, renal dysfunction, SBP, DBP, FPG, CREA, UA, TG, TC, HDL-C, and LDL-C), the ORs (95% CIs) for a CAVI ≥ 8.0 were 1.818 (1.098–3.009), and 2.346 (1.413–3.896), respectively, in Q3 and Q4 for TyG, 1.770 (1.092–2.869), and 2.434 (1.491–3.975) in Q1 and Q4 for TG/HDL-C, and 2.699 (1.235–5.897) in Q4 for METS-IR.

**TABLE 5:** Logistic regression analyses for the association between non-insulin-based IR indices and a CAVI ≥ 8.0.

Variables	OR (95%CI) for CAVI ≥ 8.0	
	Model 1	Model 2
**TG/HDL-C**		
Q1	**Reference**	**Reference**
Q2	2.151 (1.478–3.130)**	1.770 (1.092–2.869)*
Q3	1.778 (1.209–2.615)*	1.217 (0.742–1.995)
Q4	2.824 (1.964–4.060)**	2.434 (1.491–3.975)**
**TyG**		
Q1	**Reference**	**Reference**
Q2	1.933 (1.274–2.934) *	1.467 (0.874–2.461)
Q3	2.768 (1.859–4.123) **	1.818 (1.098–3.009)*
Q4	3.962 (2.698–5.818) **	2.346 (1.413–3.896)**
**METS-IR**		
Q1	**Reference**	**Reference**
Q2	1.433 (0.986–2.083)	1.571 (0.923–2.674)
Q3	2.023 (1.420–2.883)**	1.831(0.998–3.358)
Q4	2.146 (1.510–3.049)**	2.699 (1.235–5.897) *

*TG, triglyceride; HDL-C, high density lipoprotein cholesterol; TyG, triglyceride and glucose index; METS-IR, metabolic score for insulin resistance; CAVI, cardio-ankle vascular index; CI, confidence interval.*

*Model 1: unadjusted.*

*Model 2: adjusted for age, sex, BMI, smoking, drinking, prehypertension, diabetes mellitus, hyperuricemia, renal dysfunction, SBP, and DBP.*

*p < 0.05; **P < 0.001.

### Receiver Operating Characteristic Curve Analysis of Ability of Triglyceride to High-Density Lipoprotein Cholesterol Ratio, Triglyceride and Glucose Index, and Metabolic Score for Insulin Resistance to Predict Cardio–Ankle Vascular Index ≥ 8.0

**FIGURE 3:**
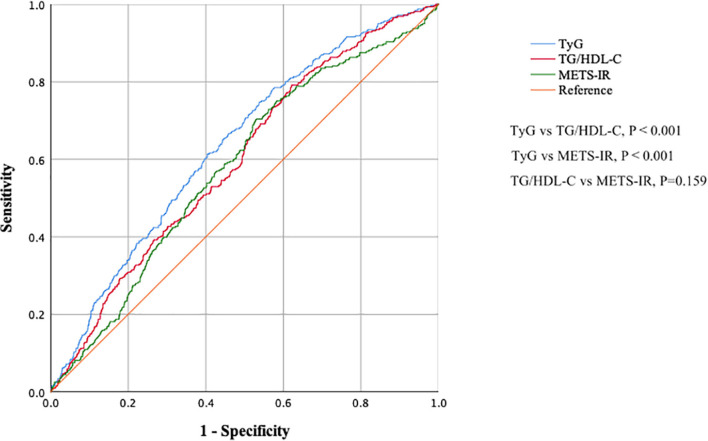
Receiver-operating characteristic curves showing the ability of TG/HDL-C, TyG, and METS-IR to detect increased arterial stiffness (CAVI ≥ 8.0). CAVI, cardio–ankle vascular index; TG/HDL-C, triglyceride to high-density lipoprotein cholesterol ratio; TyG, fasting triglyceride and glucose index; METS-IR, metabolic score for insulin resistance.

Figure 3 and Table 6 show the ROC curves for the ability of TG/HDL-C, TyG, and METS-IR to detect a CAVI ≥ 8.0. The area under the curve (AUC) was 0.598 (95% CI 0.567–0.629) for TG/HDL-C, 0.637 (0.606–0.667) for TyG, and 0.581 (0.550–0.613) for METS-IR. Hence, TyG had the highest AUC for discriminating a CAVI ≥ 8.0. The cut-off value, Youden index, sensitivity, and specificity for identification of a CAVI ≥ 8.0 was 0.600, 0.170, 0.791, and 0.379 for TG/HDL-C, 8.252, 0.210, 0.748, and 0.462 for TyG, and 30.082, 0.174, 0.704, and 0.470 for METS-IR, respectively.

**TABLE 6:** The ROC curves analysis of TG/HDL-C, TyG, and METS-IR index for discriminating CAVI ≥ 8.0.

Characteristics	TG/HDL-C	TyG	MEST-IR	*P_1_-*value	*P_2_-*value	*P_3_-*value
AUC (95%CI)	0.598 (0.567–0.629)	0.636 (0.606–0.667)	0.581 (0.550–0.613)	<0.001	0.159	<0.001
Cut-off value	0.600	8.252	30.082			
YI	0.170	0.210	0.174			
Sensitivity	0.791	0.748	0.704			
Specificity	0.379	0.462	0.470			

*AUC, area under the curve; ROC, receiver operating characteristic; TG, triglyceride; HDL-c, high density lipoprotein cholesterol; TyG, triglyceride and glucose index; METS-IR, metabolic score for insulin resistance; CI, confidence interval; YI, Youden index.*

*P1: comparison between TGı/HDL-C and TyG; P2: comparison between TGıHDL-C and METS-IR; P3: comparison between TyG and METS-IR.*

## Discussion

This cross-sectional study explored the association between TG/HDL-C, TyG, METS-IR and the risk of increased arterial stiffness and compared their discriminatory ability in non-hypertensive Chinese adults. We demonstrated a significant association between increased arterial stiffness (CAVI ≥ 8.0) and each of these three non-insulin-based IR indices. Our present findings expand on those of previously published studies by demonstrating that the discriminatory ability of TyG is better than that of TG/HDL-C or METS-IR. Therefore, TyG might be a preferable parameter for assessment of the risk of arterial stiffness in non-hypertensive subjects.

Wen et al. found that TG/HDL-C was independently associated with a baPWV > 1,400 cm/s in 1498 apparently healthy Chinese individuals who attended for routine health screening. After adjusting for conventional cardiovascular risk factors, multiple logistic regression revealed the OR of the highest quartile of TG/HDL-C for baPWV abnormalities to be 1.91 (95% CI 1.11–3.30) in men and 2.91 (95% CI 1.02–8.30) in women when compared with the lowest quartile (12). Furthermore, a positive association was found between TG/HDL-C and arterial stiffness in a study of adolescents and young adults in the United States (25), a study of postmenopausal women in Korea (26), and a study of men with diabetes in Japan (27). However, Chen and Dai found that the relationship between baPWV and TG/HDL-C was not linear, especially when the TG/HDL-C was ≥ 5.6 and when alcohol intake was excessive (28). We did not find a positive relationship between the per SD increase in TG/HDL-C and CAVI in linear regression analysis after adjusting for conventional cardiovascular risk factors. Our findings suggest a need for caution when using TG/HDL-C to detect increased arterial stiffness in view of its non-linear relationship with known arterial stiffness parameters, including baPWV and the CAVI.

METS-IR is a relatively new parameter for assessment of IR and was established by Bello-Chavolla et al. (11). More recently, Bello-Chavolla et al. found a positive relationship between PWV and non-insulin-based IR indices in a cross-sectional cohort that included 305 subjects. In that study (29), the correlation with PWV was stronger for METS-IR than for TyG or TG/HDL-C after adjusting for sex, age, antihypertensive treatment, and smoking status. However, unlike Bello-Chavolla et al., who found a linear relationship between METS-IR and arterial stiffness parameter, we found that only the Q3 and Q4 of METS-IR significantly increased the likelihood of a CAVI ≥ 8.0 when compared with the lowest quartile. This difference in findings might be attributed to the characteristics of the study participants included. For example, compared with our study population, the study by Bello-Chavolla et al. included more high-risk individuals with a higher BMI (mean 29.0 ± 5.80) (29). Compared with TyG and TG/HDL-C, METS-IR adding BMI to the formulas that are based on glucose, TG, and HDL-C increases the spectrum of explained variability of the model and might be more applicable for assessment of IR in overweight or obese subjects (11). Another potential explanation for the different findings in these two studies could lie in racial differences between the study populations. More multiethnic studies are needed in the future to explore the relationship between METS-IR and arterial stiffness.

The TyG index is a surrogate marker of IR and has been used extensively to predict hypertension and type 2 DM (30, 31). Lee et al. were the first to report that the TyG index was independently associated with increased baPWV and in a relatively large number of healthy Korean adults (32). Furthermore, a positive relationship between the TyG value and arterial stiffness has been demonstrated in Chinese patients with hypertension (13), lean postmenopausal women (14), and elderly Chinese individuals living in Beijing and Shanghai (15, 16). Wu et al. also found a significant association between the TyG value and progression of arterial stiffness in patients with hypertension during a median prospective follow-up of 4.71 years (33). In our study, linear regression analysis found a similarly positive relationship between the TyG and CAVI values in non-hypertensive subjects, in whom multiple logistic regression analysis identified an increased risk of arterial stiffness (CAVI ≥ 8.0). Of note is that a linear association between TyG and CAVI was only found in subjects older than 40 years, which suggests that TyG might perform better in the elderly population. Furthermore, our ROC curve analysis showed that the discriminatory ability of TyG was better than that of TG/HDL-C or METS-IR, which may be explained as follows. First, TyG showed a better correlation and higher predictive ability in diagnosis of IR in the Chinese population when compared with TG/HDL-C (34). Second, the METS-IR was developed based on the participants from Mexico, which might have been more accurate for evaluation of IR in this above-mentioned population (11). More research is needed to assess the clinical value of the METS-IR in evaluation of IR in the Chinese population. Even though BMI and HDL-C were included in the METS-IR formula, the difference in these parameters according to whether CAVI was ≥ 8.0 or < 8.0 was relatively weak or not significant in our study, which might also be a plausible explanation for the relatively poor performance of METS-IR in this study. Our findings suggest that the TyG could be an effective and simple parameter for assessment of the risk of arterial stiffness.

The associations between TG/HDL, TyG, METS-IR, and arterial stiffness might be attributed to IR. IR is a metabolic disorder in which there is an impaired biological response to insulin stimulation in organs and tissues, leading to poor uptake and oxidation of glucose and a decrease in glycogen synthesis (5). IR can cause hyperinsulinemia and dysfunction in endothelial cells, including activation of sodium channels, impaired synthesis of nitric oxide, activation of the renin-angiotensin-aldosterone system, and increased oxidative stress, systemic inflammation and maladaptive immune responses, leading eventually to vascular remodeling, and arterial stiffness (6, 7). Hence, use of a simple measure, such as TG/HDL, TyG, or METS-IR, to assess IR and identify individuals at high risk of arterial stiffness has important clinical value in the prevention of CVD.

This study has several strengths. First, most of the previous investigations have used baPWV to reflect arterial stiffness. Ours is only the second study to use CAVI, a relatively new arterial stiffness assessment method developed in 2004, for investigation of the correlation between arterial stiffness and non-insulin-based indices. CAVI, which reflects arterial stiffness from the origin of the aorta to the ankle (17), is not affected by changes in BP and is superior to baPWV for assessment of arterial stiffness (18). Therefore, our findings provide more clinical evidence to support use of non-insulin-based indices for evaluation of the risk of arterial stiffness. Second, we excluded patients with hypertension and those taking hypoglycemic and lipid-lowering medication. Therefore, these non-insulin-based indices could also be used in populations with a low CVD risk and those attending health management centers. Third, to the best of our knowledge, this study is the first to compare TG/HDL, TyG, and METS-IR in terms of their ability to identify arterial stiffness. We demonstrated that TyG performed better than TG/HDL and METS-IR for identification of the risk of arterial stiffness in non-hypertensive subjects.

Our study also had some limitations. First, it had a cross-sectional design, which meant that the causal relationships between the non-insulin-based IR indices and arterial stiffness could not be clearly determined. More prospective cohort studies are needed to reveal the relationship between TG/HDL-C, TyG, and METS-IR and the incidence of arterial stiffness. Second, only Han Chinese individuals were studied. Therefore, our findings may not be generalizable to other ethnic populations. Third, owing to the relatively large sample size and high cost of testing, we did not measure plasma insulin concentration in this cohort. Fourth, the study data were obtained from a health management center for a period that was 7 years earlier, when dietary habits and physical activity participants were not often recorded. Therefore, we could not adjust for these factors in the multiple logistic regression analyses.

## Conclusion

We have demonstrated significant associations between increased arterial stiffness and non-insulin-based IR indices, including TG/HDL-C, TyG, and METS-IR. We found that TyG had the best ability to detect arterial stiffness. Therefore, TyG might be a preferable parameter for assessment of the risk of arterial stiffness for non-hypertensive Chinese subjects.

## Data Availability Statement

The original contributions presented in this study are included in the article/supplementary material, further inquiries can be directed to the corresponding authors.

## Ethics Statement

The studies involving human participants were reviewed and approved by the Medical Ethics Committee of West China Hospital, Sichuan University. The patients/participants provided their written informed consent to participate in this study. Written informed consent was obtained from the individual(s) for the publication of any potentially identifiable images or data included in this article.

## Author Contributions

XZ and RY designed the study’s intellectual content and wrote this initial manuscript. CY participated in the original data acquisition and literature search. XZ, RY, and TL undertook the statistical analysis and participated in manuscript preparation. XC and TL revised the manuscript for important intellectual content and languages. All authors read and approved the final manuscript.

## Conflict of Interest

The authors declare that the research was conducted in the absence of any commercial or financial relationships that could be construed as a potential conflict of interest.

## Publisher’s Note

All claims expressed in this article are solely those of the authors and do not necessarily represent those of their affiliated organizations, or those of the publisher, the editors and the reviewers. Any product that may be evaluated in this article, or claim that may be made by its manufacturer, is not guaranteed or endorsed by the publisher.

## Abbreviations

IR: Insulin resistance
CAVI: Cardio–ankle vascular index
TG/HDL-C: Triglyceride to high-density lipoprotein cholesterol ratio
TyG: Fasting triglyceride and glucose index
METS-IR: Metabolic score for IR
ROC: Receiver operating characteristic
CVD: Cardiovascular disease
baPWV: brachial–ankle PWV
cfPWV: carotid–femoral PWV
BP: Blood pressure
SBP: Systolic blood pressure
DBP: Diastolic blood pressure
FPG: Plasma fasting glucose
TG: Triglyceride
TC: Total cholesterol
HDL-C: High-density lipoprotein cholesterol
LDL-C: Low-density lipoprotein cholesterol
UA: Uric acid
CREA: Creatinine
DM: Diabetes mellitus
HUA: Hyperuricemia
RD: Renal dysfunction
BMI: Body mass index
IQR: Interquartile range
AUC: Area under curve
SD: Standard deviation
CI: Confidence interval
OR: Odds ratio.
